# Diagnostic accuracy of fecal calprotectin in predicting significant gastrointestinal diseases

**DOI:** 10.1002/jgh3.12548

**Published:** 2021-05-06

**Authors:** Yee Man Kan, Sin Yan Chu, Ching Kong Loo

**Affiliations:** ^1^ Department of Medicine and Geriatrics Kwong Wah Hospital Kowloon Hong Kong; ^2^ Department of Pathology Kwong Wah Hospital Kowloon Hong Kong

**Keywords:** colon cancer, colonic neoplasms, colonoscopy, fecal calprotectin, functional disorders, gastroenterology

## Abstract

**Background and Aim:**

It is often unreliable to triage patients for timely endoscopic investigations based on symptoms alone. We need an objective assessment to differentiate between organic gastrointestinal diseases and functional bowel symptoms. We evaluated the diagnostic accuracy of fecal calprotectin (FC) in predicting organic gastrointestinal diseases.

**Methods:**

In a prospective observational study, consecutive patients referred for colonoscopy to the Department of Medicine and Geriatrics at the Kwong Wah Hospital in Hong Kong were recruited. Stool samples were collected within 24 h before colonoscopy. FC was measured by a commercial kit. Upper endoscopy investigations were then proceeded if normal colonoscopy but elevated FC.

**Results:**

Two hundred and seventy out of 429 patients had FC above 50 μg/g. Eighty‐six out of 270 with elevated FC had significant colonoscopy pathological findings. The sensitivity, specificity, positive predictive value (PPV), and negative predictive value (NPV) of FC test for diagnosing a significant organic colonoscopy or upper endoscopy disease were 91.7, 55.6, 57.0, and 91.2%, respectively. The NPV of FC for colorectal cancer, high risk polyp, and colon inflammation were 98.7, 96.2, and 98.1%, respectively. The NPV of FC in the condition of altered bowel habit or abdominal pain in predicting colorectal cancer and inflammation were 93.8 and 100%, respectively.

**Conclusions:**

FC is a reliable marker of ruling out organic bowel diseases. A single negative FC test could be used as a triage tool to prioritize the need and urgency of further investigation, particularly in the setting of altered bowel habits and abdominal pain.

## Graphical abstract

A negative fecal calprotectin test could rule out significant colorectal diseases, particularly in the clinical setting of change of bowel habit and recurrent abdominal pain. Fecal calprotectin can be a reliable triage tool to prioritize colonoscopy.

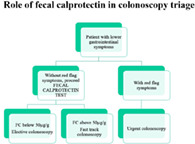

## Introduction

Patients presenting with gastrointestinal symptoms can be difficult to assess.^1^ Some features, for example, rectal bleeding, a mass on examination, iron deficiency anemia might suggest serious pathology, but nonspecific symptoms such as a persistent change in bowel habit might also be the presentation of a significant problem. The National Institute for Health and Care Excellence (NICE) concedes that symptoms have a positive predictive value (PPV) for colorectal cancer (CRC) of only 3–4%.^2^ A meta‐analysis also concluded that symptoms alone are poor predictors of underlying pathology.^3^ In the absence of any reliable predictor of significant pathology, all patients with bowel symptoms have to be referred to colonoscopy. However, colonoscopy is invasive and expensive. A substantial number of patients with bowel symptoms actually suffer from nonorganic diseases, for example, irritable bowel disorders.^4^ In other words, colonoscopy might not be necessary in this group of patients. Therefore, a reliable, noninvasive and easily measured test is advocated to be the triage tool to guide who requires early colonoscopy.

Fecal calprotectin FC is a cytosolic protein in neutrophil granulocytes. It correlates with neutrophilic infiltration of the intestinal mucosa.^5^ It has been investigated as a biological marker of intestinal inflammation.^6^ Fecal calprotectin can reliably distinguish inflammatory bowel disease from functional gastrointestinal disorder, and correlates well with inflammatory bowel disorder disease activity.^7^, ^8^ Increased levels have also been described in colorectal neoplasia, microscopic colitis, and bacterial diarrhea.^9^, ^10^, ^11^ Calprotectin is also resistant to enzymatic degradation during passage through the gastrointestinal tract and remains stable up to 7 days at room temperature.^12^ It is easily measurable as well. Fecal calprotectin level can now be determined with a commercially available quantitative enzyme‐linked immunoassay. The final result can be available after 15 min of processing. A value below 50 μg/g is considered normal.

Increased levels of fecal calprotectin have been reported in patients with several inflammatory conditions of the lower gastrointestinal tract and even in patients affected by neoplasm of both the upper and the lower gastrointestinal tract.^13^, ^28^ Therefore, measuring fecal calprotectin has been proposed as a useful noninvasive diagnostic tool for differentiating patients with organic disease of the intestinal tract from those with functional diseases.^14^ Testing fecal calprotectin level offers the possibility of safer and more rapid diagnosis of the absence of clinically significant bowel diseases, minimizing patient anxiety, inconvenience, and exposure to unnecessary prolonged‐waiting colonoscopies.^15^

The aim of this study was, therefore, to prospectively investigate the diagnostic accuracy of fecal calprotectin in predicting significant gastrointestinal pathology.

## Methods

### Setting and participants

This was a prospective observational study, recruiting patients in Department of Medicine and Geriatrics of Kwong Wah Hospital, referred for colonoscopic examination for various indications, including screening.

Exclusion criteria were pregnancy, concomitant serious illness, and evidence of acute respiratory tract infection.

Clinical data and endoscopic findings were collected by experienced endoscopists. Indications for colonoscopy were also recorded. The endoscopists were blinded to the fecal calprotectin results.

Significant colonoscopy findings included malignancy CRC, colitis, high‐risk polyp (HRP) (polyp greater than 1 cm in diameter, polyp of high grade dysplasia, polyp of villous/serrated features, multiple polyps ≥3), and bleeding lesions. Patients with no significant colonoscopy finding but elevated fecal calprotectin levels (>50 μg/g) were further investigated with upper endoscopy. Significant upper endoscopy findings included severe inflammation, which showed the presence of mucosal breaks or erosions, peptic ulcer disease, malignancy, and bleeding. The endoscopists performing the follow‐up endoscopy were aware of the reason for the investigation (positive fecal calprotectin test).

The pathologists who examined the biopsies obtained at endoscopy were also blinded to the fecal calprotectin levels.

### Measurement of fecal calprotectin

Patients were asked to collect a fecal sample the day before the colonoscopy preparation. They were asked to bring the samples to endoscopy admission ward on the day of the procedures. Fecal samples were tested for calprotectin at a single laboratory by means of a commercially available kit (BÜHLMANN Quantum Blue). All fecal samples were expected to be processed within 72 h after collection. The laboratory personnel who were responsible for handling these specimens were blinded to the patients' clinical history and endoscopic findings.

### Statistical analysis

Results are shown as mean. Test characteristics are presented as sensitivity, specificity, and positive and negative predictive values.

## Results

### Patients' characteristics

Four hundred and twenty‐nine adult Chinese patients (males 210, mean age 62.5 years old, range 23–89) were recruited in this study. Their fecal specimens were collected the day before colonoscopy studies. Those of normal colonoscopy findings but elevated fecal calprotectin FC result above 50 μg/g underwent upper endoscopy. Indications for colonoscopy are summarized in Table 1. Table 2 shows the colonoscopy diagnoses.

**Table 1:** Indications for colonoscopy in recruited patients

Indication for colonoscopy	Number
Surveillance (either colorectal cancer screening or after removal of colorectal cancer or polyps)	66
Rectal bleeding (either overt or occult)	84
Abdominal pain	22
Change of bowel habit	105
Anemia	116
Constipation	21
Weight loss	1
Elevated CEA	3
Other indications	11
Total	429

CEA, Carcinoembryonic Antigen.

**Table 2:** Colonoscopy diagnosis in recruited patients

Colonoscopy diagnosis	Number
Normal finding/uncomplicated hemorrhoids/diverticulosis	225
Colorectal cancer	17
Non‐high‐risk colon polyp	107
High‐risk polyp	35
Mild inflammation	3
Significant inflammation	36
Angiodysplasia	4
Neuroendocrine tumor	2
Total	429

Colonoscopy was normal in 225 patients. Nineteen cases of CRC were diagnosed (13 colon cancer, 2 rectal cancer, 2 intramucosal adenocarcinoma, and 2 neuroendocrine tumor). Colon polyps of low risk were diagnosed in 107 patients. Thirty‐five cases of HRPs were identified. Active colon mucosal inflammation was identified in 39 patients (3 mild inflammation and 36 significant inflammatory activities).

Two hundred and seventy of 429 patients had fecal calprotectin values above 50 μg/g. Eighty‐six of 270 with elevated fecal calprotectin results had significant colonoscopy pathological findings. Seventy‐four patients with elevated fecal calprotectin results had significant upper endoscopy pathological findings but normal colonoscopy studies. They were 1 gastric adenocarcinoma, 2 candida esophagitis, 1 bleeding gastric antral vascular ectasia, 3 inflammatory gastric polyps, 9 LA class C or D esophagitis +/− esophageal ulcer, 34 peptic ulcer disease, and 24 severe/hemorrhagic gastritis or duodenitis with mucosal breaks or erosions. One patient with rectal cancer and one patient with intramucosal adenocarcinoma had normal fecal calprotectin result. Table 3 shows the symptom prevalence for CRC, HRP, and colon mucosal inflammation in patients referred for colonoscopy.

**Table 3:** Symptom prevalence for colorectal cancer (CRC), high‐risk polyp (HRP), and colitis in patients referred for colonoscopy

Prevalence of symptoms	Total Number	%	CRC Number	%	HRP Number	%	Inflammation Number	%	CRC + HRP+ inflammation Number	%
Rectal bleeding	84	19.6	4	21.1	6	17.1	9	23.1	21	21.6
Anemia	116	27.0	13	68.4	15	42.9	7	17.9	36	37.1
Chronic diarrhea	6	1.4	0	0	0	0	3	7.7	3	3.1
Altered bowel habit	105	24.5	1	5.3	9	25.7	1	2.6	11	11.3
Constipation	21	4.9	0	0	1	2.9	0	0	2	1.0
Abdominal pain	22	5.1	1	5.3	1	2.9	2	5.1	4	4.1
Surveillance	66	15.4	0	0	3	8.6	14	35.9	17	17.5
Weight loss	1	0.23	0	0	0	0	0	0	0	0
Elevated serum CEA	3	0.7	0	0	0	0	0	0	0	0

CEA, Carcinoembryonic Antigen.

As a consequence, the sensitivity and specificity of fecal calprotectin test in predicting a significant colonoscopy finding were 88.7 and 44.6%, respectively. Positive predictive value (PPV) and negative predictive value (NPV) for predicting significant colonoscopy were 31.9 and 93.1%, respectively. The corresponding figures for diagnosing a significant organic colonoscopy or upper endoscopy disease were respectively 91.7, 55.6, 57.0, and 91.2% when patients with upper endoscopy performed were included.

We then analyzed the diagnostic values of fecal calprotectin test in predicting CRC, HRP, and colon inflammation (Table 4). The sensitivity, specificity, PPV, and NPV of an elevated fecal calprotectin level for diagnosing CRC were 89.5, 38.3, 6.3, and 98.7%, respectively, while for diagnosing the presence of HRP were 82.9, 38.8, 10.7, and 96.2%, respectively. For diagnosing colon mucosal inflammation, they were respectively 92.3, 40, 13.3, and 98.1%.

**Table 4:** Performance of fecal calprotectin test in the detection of colorectal cancer (CRC), high‐risk polyp (HRP), and mucosal inflammation

	CRC	HRP	Inflammation	CRC + HRP + inflammation+ bleeding lesions^†^
Number of cases	19	35	39	97
Sensitivity	89.5%	82.9%	92.3%	88.7%
Specificity	38.3%	38.8%	40.0%	44.6%
PPV	6.3%	10.7%	13.3%	31.9%
NPV	98.7%	96.2%	98.1%	93.1%

^†^ Bleeding lesions, for example, angiodysplasia, bleeding diverticulosis.

NPV, negative predictive values; PPV, positive predictive values.

Further, we tried to specify the diagnostic values of elevated fecal calprotectin according to the indications of colonoscopy (Table 5).

**Table 5:** Diagnostic value of fecal calprotectin test in different clinical conditions with colonoscopy performed

	Anemia		Rectal bleeding		Chronic diarrhea		Altered bowel habit		constipation		Abdominal pain		Surveillance	
	+ CLN	+ENDO	+ CLN	+ENDO	+ CLN	+ENDO	+ CLN	+ENDO	+ CLN	+ENDO	+ CLN	+ENDO	+ CLN	+ENDO
Sensitivity	86.1%	90.4%	95.2%	91.2%	100%	100%	72.7%	90.3%	100%	100%	100%	100%	88.2%	92.3%
Specificity	42.5%	52.4%	39.7%	46%	33.3%	50%	47.9%	60.8%	42.1%	50%	44.4%	61.5%	51.0%	62.5%
PPV	40.3%	61.0%	34.5%	53.4%	60.0%	80%	14.0%	49.1%	11.5%	38.5%	28.6%	64.3%	38.5%	61.5%
NPV	87.2%	84.6%	96.2%	88.5%	100%	100%	93.8%	93.8%	100%	100%	100%	100%	92.6%	92.6%

+CLN = significant colonoscopy findings; + ENDO = either significant upper endoscopy or colonoscopy findings.

NPV, negative predictive values; PPV, positive predictive values.

Among 116 patients referred for investigation of anemia, 13 CRC, 15 HRP, and 7 colon mucosal inflammation were found. Fecal calprotectin levels were elevated in 12 out of 13 patients with CRCs. Two patients with HRP had fecal calprotectin below cutoff level, as well as two patients with colon inflammation. In this subgroup of patients, sensitivity and specificity of elevated fecal calprotectin level for significant colonoscopy findings were 86.1 and 42.5%, respectively. PPV and NPV were 40.3 and 87.2%, respectively. Including those with significant upper endoscopy findings, the sensitivity, specificity, PPV, and NPV of elevated fecal calprotectin level in this group of patients were 90.4, 52.4, 61.0, and 84.6%, respectively.

Among 84 patients referred for rectal bleeding, 4 were found to have CRC, 6 HRP, and 9 colon mucosal inflammation. Three of 4 patients with CRC had elevated fecal calprotectin level while all of those with HRP and mucosal inflammation in this group had fecal calprotectin above the cutoff level. Therefore, sensitivity and specificity of elevated fecal calprotectin level for significant colonoscopy findings were 95.2 and 39.7%, respectively. PPV and NPV were 34.5 and 96.2%, respectively. Including those with significant upper endoscopy findings, the sensitivity, specificity, PPV, and NPV of elevated fecal calprotectin level in this group of patients were 91.2, 46, 53.4, and 88.5%, respectively.

Among 105 patients referred for investigation of altered bowel habits, one CRC, nine HRP, and one colon mucosal inflammation were found. Fecal calprotectin levels were elevated in these patients with CRC or colon mucosal inflammation. In this subgroup of patients, sensitivity and specificity of elevated fecal calprotectin level for significant colonoscopy findings were 72.7 and 47.9%, respectively. PPV and NPV were 14.0 and 93.8%, respectively. Including those with significant upper endoscopy findings, the sensitivity, specificity, PPV, and NPV of elevated fecal calprotectin level in this group of patients were 90.3, 60.8, 49.1, and 93.8%, respectively.

Among 22 patients referred for abdominal pain, all found to CRC, HRP, or colon inflammation had elevated fecal calprotectin levels. In this subgroup of patients, sensitivity and specificity of elevated fecal calprotectin level for significant colonoscopy findings were 100 and 44.4%, respectively. PPV and NPV were 28.6 and 100%, respectively. Including those with significant upper endoscopy findings, the sensitivity, specificity, PPV, and NPV of elevated fecal calprotectin level in this group of patients were 100, 61.5, 64.3, and 100%, respectively.

For those who were referred for CRC screening, surveillance of colorectal polyps or inflammatory bowel diseases, symptoms of constipation or diarrhea, the diagnostic values of FC in these clinical conditions are summarized in Table 5.

## Discussion

Fecal calprotectin level has been established to be an adjunctive tool reflecting the activity of inflammatory bowel disease.^5^, ^16^, ^17^, ^18^, ^19^ In recent years, fecal calprotectin has been proposed to be a biological marker of organic gastrointestinal diseases.^20^, ^21^, ^22^ It has been advocated to be a cost‐effective triage tool for making timely referral of colonoscopy in primary care.^15^, ^23^ A meta‐analysis revealed that fecal calprotectin was clinically useful for distinguishing organic bowel diseases from functional disorders.^24^

Our data show that among patients referred for colonoscopy, fecal calprotectin levels below 50 μg/g have only a small risk for large bowel organic disease. The NPV of FC was 93.1% We have further investigated the diagnostic values of FC according to the findings of colonoscopy. Their sensitivity and NPV ranged from 82 to 92% and 96 to 98%, respectively. Thus, FC below 50 μg/g could effectively exclude the possibility of underlying CRC, HRP, and significant colon inflammation.

Elevated calprotectin levels were found in 57.3% of patients with normal colonoscopy and in 51.4% of those with trivial endoscopic findings such as uncomplicated diverticulosis or low‐risk polyps. These data are similar to those from previous prospective studies in which elevated FC levels were found in 30–60% of normal population.^25^, ^26^, ^27^ However, the possibility of having upper gastrointestinal tract lesions could not be definitely ruled out in some of the patients with elevated FC but normal colonoscopy.^13^, ^28^ Therefore, patients with no significant colonoscopy finding but elevated FC levels (>50 μg/g) were further investigated with upper endoscopy in our study. Seventy‐four out of 151 had elevated FC results but normal colonoscopy had significant upper endoscopy findings. By including patients of elevated FC levels but normal colonoscopy, the sensitivity and specificity of FC increased from 88.7 to 91.7% and 44.6 to 55.6%, respectively. The PPV increased from 31.9 to 57.0%. The NPV remained higher than 90%.

For some clinical conditions, we have identified that a normal fecal calprotectin level may be helpful in ruling out a diagnosis of significant colorectal disease. Among 127 patients referred for altered bowel habits or abdominal pain, 6 were found to have CRC or colon inflammation. The FC levels were elevated in all of them. Therefore, the NPV of FC in the condition of altered bowel habit or abdominal pain in predicting CRC or inflammation was up to 100%.

In contrast, our data suggested that the PPV of predicting significant colonoscopy was only 31.9%. The PPV and NPV increased to 57.0 and 91.2%, respectively, if both colonoscopy and upper endoscopy findings were counted. This study demonstrates that in unselected patients referred for colonoscopy, a single assessment of FC may not be sufficiently accurate to identify those with significant colorectal disease. However, a normal result can help in ruling out organic disease among patients with altered bowel habits or abdominal pain. We confirm findings of prior studies that measurement of FC before consideration of further costly endoscopic investigations provided valuable diagnostic assistance in the setting of altered bowel habit or abdominal pain, without clinical alarming features.^4^, ^14^, ^27^, ^29^, ^30^, ^31^, ^32^

There are several limitations in this study. Firstly, small bowel lesions had not been assessed. For those with anemia, chronic diarrhea, or abdominal pain, FC level might not be normal in certain organic small bowel disorders, such as small bowel tumors or small bowel Crohn's disease.^33^, ^34^, ^35^ Secondly, 28 patients refused to proceed upper endoscopy or defaulted it. The adjusted diagnostic value of FC might be influenced if these patients were counted. Thirdly, there were quite a number of patients taking medication, which might cause falsely positive FC.^36^, ^37^, ^38^ Overall, 30 patients had exposure to nonsteroidal anti‐inflammatory drugs NSAID or systemic steroid, and 111 patients were proton pump inhibitor PPI users. One hundred and thirteen patients were taking antiplatelet agents. Nineteen patients (7 warfarin, 12 direct oral anticoagulant) took anticoagulants at least 3 days before colonoscopy. Prior studies suggested that significant higher levels of FC were found in patients taking NSAID, steroid, antiplatelet agents, anticoagulants, or PPI. It might explain why the PPV of an abnormal colonoscopy finding was low, only 31.9%. The concurrent use of antiplatelet agents, oral anticoagulant, oral steroid, and NSAID might result false positive FC test.

We conclude that fecal calprotectin can be a reliable marker for ruling out organic bowel diseases. A single negative FC test could enable an objective assessment of the need and urgency of further investigation, particularly in the setting of altered bowel habits and abdominal pain. Therefore, people with these symptoms and negative FC tests could be spared from expensive and rather invasive colonoscopy tests. FC test could be used as a triage tool to prioritize endoscopy service. A negative FC result could help to relieve patient's anxiety as well. The implementation of it in clinical practice might help relieve the burden of our overwhelming health care system, particularly during the era of COVID‐19 pandemic when the access to diagnostic endoscopy across different nations has dramatically reduced. However, positive result should be investigated further. Large‐scale prospective studies in the analysis of its clinical cost‐effectiveness are warranted.
